# The Role of Oxidative Stress on the Pathogenesis of Graves' Disease

**DOI:** 10.1155/2012/302537

**Published:** 2011-12-10

**Authors:** Miloš Žarković

**Affiliations:** ^1^School of Medicine, University of Belgrade, 11000 Belgrade, Serbia; ^2^Clinic of Endocrinology, Clinical Center of Serbia, Dr Subotića 13, 11000 Belgrade, Serbia

## Abstract

Graves' disease is a most common cause of hyperthyroidism. It is an autoimmune disease, and autoimmune process induces an inflammatory reaction, and reactive oxygen species (ROSs) are among its products. When balance between oxidants and antioxidants is disturbed, in favour of the oxidants it is termed “oxidative stress” (OS). Increased OS characterizes Graves' disease. It seems that the level of OS is increased in subjects with Graves' ophthalmopathy compared to the other subjects with Graves' disease. Among the other factors, OS is involved in proliferation of orbital fibroblasts. Polymorphism of the 8-oxoG DNA N-glycosylase 1 (hOGG1) involved in repair of the oxidative damaged DNA increases in the risk for developing Grave's disease. Treatment with glucocorticoids reduces levels of OS markers. A recent large clinical trial evaluated effect of selenium on mild Graves' ophthalmopathy. Selenium treatment was associated with an improved quality of life and less eye involvement and slowed the progression of Graves' orbitopathy, compared to placebo.

## 1. Introduction

Graves' disease is a most common cause of hyperthyroidism in iodine sufficient areas [1]. It is characterized by diffuse goitre and hyperthyroidism. Graves' orbitopathy represents orbit involvement and is clinically relevant in about half of the patients with the Graves' disease. In 3 to 5% of the patients, orbitopathy is severe [2]. Graves' disease is an autoimmune disease characterized by the presence of the serum autoantibodies. TSH receptor antibody represents the major autoantibody in Graves' disease [3].

Autoimmune process induces an inflammatory reaction and reactive oxygen species (ROSs) are among its products. ROSs are formed as normal metabolic products and are important in normal cellular functioning, but their production can be increased under pathological conditions and cause damage [4, 5]. Therefore, a large number of antioxidant systems act as protective mechanism. Among them are superoxide dismutase which catalyses dismutation of superoxide to peroxide, catalase which catalyses the decomposition of hydrogen peroxide to water and oxygen, while glutathione peroxidise which reduces lipid hidroperoxides while simultaneously oxidizing glutathione [6]. Situation in which balance between oxidants and antioxidants is disturbed in favour of the oxidants is termed “oxidative stress” (OS) [4].

## 2. Oxidative Stress and the Thyroid Gland

Synthesis of thyroid hormones requires formation of the hydrogen peroxide, a highly reactive oxidant. Hydrogen peroxide and oxidized iodine are immediately used in peroxidation reaction that is catalysed by thyroid peroxidase [7]. To protect thyroid cells from reactive oxygen species (ROSs) a potent antioxidant system exists in thyroid. Peroxiredoxin, glutathione peroxidase, thioredoxin, and catalase are involved in this antioxidant system [8]. Peroxiredoxins belong to a family of antioxidant proteins that are well conserved during evolution. Peroxiredoxin 5 (PRDX5) is expressed in the thyroid, mostly in the cytoplasm. The level of expression is correlated with the functional status of thyroid cells, being higher in multinodular goitres, and even higher in hyperthyroid tissues [9]. Catalase and glutathione peroxidases are also increased in hyperthyroid tissues [10]. 

Some level of oxidative load is necessary for thyroid function and proliferation. In a healthy thyroid, ROSs are produced in an area that is located at the apical pole of the cell in microvilli, where H_2_O_2_ is consumed either during the hormone synthesis or by antioxidant systems. However, Th1-induced ROS production causes ROS accumulation both in the cytoplasm and in nuclei, where it can become toxic. Interestingly, *in vivo*, both the antioxidant N-acetylcysteine (NAC) and the anti-inflammatory prostaglandin 15deoxy-12,14-prostaglandin J2 (15dPGJ2) protect the thyroid against toxic effects of the OS. It seems that NAC and 15dPGJ2 mainly act on infiltrating inflammatory cells, reducing the extrafollicular ROS load [11]. As hydrogen peroxide and iodine are cosubstrates in thyroid hormone production, iodine inhibits hydrogen peroxide production [12]. Tobacco smoke contains thyocyanate that blocks iodine transport into thyrocite. This could increase H_2_O_2_ production and oxidative load, especially when associated with other environmental factors [13, 14].

Poncin and coworkers suggested that thyroid interstitial inflammation depends on the balance of the OS and the antioxidative defences (AODs). In basal, healthy conditions, both OS and AOD are low, and there is no inflammation. Increase in OS balanced by the increase in AOD would lead to minimal inflammation, but unopposed increase in OS would lead to strong inflammation and cell necrosis. Reducing OS would lead to inflammation reduction and vice versa [11, 15].

## 3. Oxidative Stress in Graves' Disease and Peripheral Tissues

Graves' disease is characterized by increased oxidative stress. Abalovich at all found increased markers of OS and decrease in markers of AOD in erythrocytes of patients with Graves' disease. All analysed markers normalized when euthyroidism was achieved after treatment with methimazole. However, after treatment with radioactive iodine, levels of tert-butyl hydroperoxide initiated chemiluminiscence and superoxide dismutase levels did not normalize [16]. Increased markers of OS were found in plasma of Graves' disease patients, even when they are rendered euthyroid. Levels of OS and AOD markers were higher, both in plasma and in thyroid tissue in patients whose treatment was shorter than 6 months [17]. However, thyroid hormones, per se, induce OS, which is tissue and species specific [18]. Even in subclinical hyperthyroidism, oxidative stress and antioxidative response seem to be increased [19]. It seems that the oxidative stress-induced activation of the NF-kappaB pathway might play a role in the autoimmune response in hyperthyroidism [20, 21]. Therefore, when antioxidant supplementation is added to methimazole, euthyroidism is more rapidly achieved [22]. However, it seems that the level of OS is increased in subjects with Graves' ophthalmopathy compared to the other subjects with the Graves' disease. Methimazole treatment normalizes markers of oxidative stress in plasma in subject with Graves' disease, but not in subjects with Graves' ophthalmopathy [23].

Hyperthyroidism is associated with increased lipid peroxidation products in rat liver and with increased activities of glutathione peroxidase, superoxide dismutase, and catalase in the liver [24]. Liver oxidative stress increases quickly after increase of thyroid hormones [25]. In rat kidney and testis, hyperthyroidism is associated with increased oxidative stress and lipid peroxidation [26–28].

Hyperthyroidism is also associated with increased oxidative stress and oxidative damage to lipids and genomic DNA in the aortic wall [29]. During hyperthyroidism, there is an increase in myocardial oxidative stress that is associated with lipid peroxidation and protein oxidation. Myocardial antioxidant enzyme activities elevation accompanied by protein expression induction occurs after four weeks of hyperthyroidism [30]. It seems that oxidative stress plays an important role in cardiac hypertrophy, by the redox activation of AKT1 and JUN/FOS signaling pathways [31]. Redox imbalance due to hyperthyroidism induces adaptation of antioxidant systems, also inducing ERK1/2 activation and leading to development of cardiac hypertrophy [32]. It is interesting to note that although long-term thyroxin administration causes cardiac hypertrophy, it is also associated with enhanced tolerance of the myocardium to ischemia and reperfusion. This response may involve the thyroid hormone-induced upregulation of HSP70 [33]. In skeletal muscle, hyperthyroidism causes increased oxidative stress associated with oxidative modification in myosin heavy chain causing the decrease in force production [34].

## 4. Oxidative Stress in Graves' Disease and Retroorbital Tissues

Graves' orbitopathy is caused by inflammation in the orbital connective tissue. Enhanced adipogenesis and overproduction of glycosaminoglycans causes an increase in orbital volume and fibrosis of the extraocular muscles [35]. Among the other factors, OS is involved in proliferation of orbital fibroblasts. In orbital fibroblasts, obtained from subjects with severe grave orbitopathy, superoxide radicals induce a dose-dependent cellular proliferation. This effect is not observed in fibroblast cultures obtained from control subjects [36]. However, superoxide-induced fibroblast proliferation could be prevented by methimazole, the xanthine oxidase inhibitor allopurinol, and nicotinamide [36, 37]. In orbital tissue samples, there is increased level of lipid hydroxyperoxide, superoxide dismutase, glutathione peroxidise, and glutathione reductase in Graves' orbitopathy patients, compared to controls. Furthermore, there is strong negative correlation between the ophthalmopathy index and glutathione level [38]. 

IL-1*β* is produced by activated macrophages and is an important mediator of the inflammatory response. Adding IL-1*β* to cultures of retroorbital fibroblasts causes an increased oxygen-free radical production in a dose-dependent manner. This is observed both in Graves' and in control cultures. Total intracellular superoxide dismutase (SOD) activity was stimulated by IL-1*β*, both in control and in Graves' cultures. However, in Graves' cultures SOD activity was increased at rest and less responsive to IL-1*β* stimulation. IL-1*β* was a potent stimulator of glycosaminoglycan (GAG) accumulation in both normal and GO retroocular fibroblasts. IL-1*β* significantly stimulated the GAG synthesis in both normal and Graves' fibroblasts cells in a dose-dependent manner. Adding SOD and catalase partially blocked accumulation of the GAG induced by IL-1*β* [39].

HSP72 is a stress inducible form of cytosolic HSP70. Its expression is induced by the environmental stress, such as heat shock, anoxia, and ischemia. HSP72 has cytoprotective effects and functions as a molecular chaperone in protein folding, transport, and degradation. Moreover, HSP72 can inhibit apoptosis by several different mechanisms. In addition, HSPs are potent activators of the innate immune system and they stimulate the production of proinflammatory cytokines. In retroorbital fibroblasts obtained from GO patients, both H_2_O_2_ and heat stress significantly increased HSP72 expression. Antioxidants, methimazole, and PTU reduced H_2_O_2_-induced HSP72 expression, and to a lesser degree heat-induced HSP72 expression [40–42].

Oxidative DNA damage was found to be significantly elevated in cultured orbital fibroblasts, but only slightly increased in fibroadipose tissues of patients with Graves' orbitopathy. In patients with Graves' orbitopathy, there was significant correlation between TSH receptor antibody levels and 8-hydroxy-2′-deoxyguanosine (a biomarker of DNA damage) content [43]. The presence of oxidative stress parameters in cultured orbital fibroblasts and its correlation with TSH receptor antibody levels represents a good indication that oxidative stress exerts action in GO. 

Urinary 8-hydroxy-2′-deoxyguanosine (8-OhdG) is also a marker of oxidative DNA damage. The study by Tsai et al. found that the urinary level of 8-OHdG was significantly increased in GO patients (1.9-fold compared with normal subjects). This increase was pronounced in patients with active GO (2.4-fold compared with normal subjects). Moreover, urinary 8-OhdG level significantly correlated with both clinical activity score and ophthalmopathy index. However, this association becomes nonsignificant after adjustment for other parameters, particularly the smoking status. It should be noted that smoker had higher urinary 8-OhdG level than never-smokers, and that smoking was significant factor in multivariate analysis [44]. It is well known, from epidemiological studies, that strong evidence for a causal association between smoking and development of Graves' orbitopathy exists [45]. Study by Tsai et al. implies that smoking-induced oxidative stress contributes to the pathogenesis of Graves' orbitopathy [44].

One of the major forms of DNA damage induced by OS is 7, 8-dihydro-8-oxoguanine, referred in an abbreviated way as 8-oxoguanine (8-oxoG). This type of DNA damage is repaired by the base excision repair pathway. This pathway is initiated by the recognition and excision of the oxidized guanine by a DNA glycosylase. In humans, the major glycosylase is 8-oxoG DNA N-glycosylase 1 (hOGG1). The hOGG1 is located on chromosome 3p25/26 and is highly polymorphic. The C to G substitution at position 1245 in exon 7 results in substitution of serine with cysteine in codon 326 has been associated with a reduced capacity to repair oxidative DNA damage. Tanrikulu et al. assessed hOGG1 Ser326Cys polymorphism (rs1052133) as a candidate risk factor for GD. They found that Cys/Cys genotype had a 3.5-fold (95% CI: 2.10–6.01, *P* < 0.001) and the Cys allele had 1.83-fold (95% CI: 1.43–2.34, *P* < 0.001) increase in the risk for developing Grave's disease in their population [46]. The Ser326Cys polymorphism in hOGG1 gene was shown to reduce the hOGG1 activity in both *in vitro* and *in vivo* studies [46]. As the production of 8-oxoG is increased both in retroorbital fibroblasts and in urine of patients with GD and correlates with the disease activity, it could be argued that reduced hOGG1 activity causes increased DNA damage and increased OS making subject more susceptible to development of Graves' orbitopathy [43, 44].

## 5. Antioxidants as Treatment for Graves' Disease

Treatment of the Graves' disease reduces OS both by rendering patients euthyroid and by the direct effect of antithyroid drugs, particularly methimazole, on OS. Methimazole completely normalized parameters of OS in peripheral erythrocytes, while radioactive iodine did not [16]. In cultured fibroblasts methimazole prevented superoxide-induced fibroblast proliferation, while propylthiouracil had little effect [36]. Other forms of treatment for Graves' disease also influence parameters of OS. In euthyroid patients treatment of Graves' ophthalmopathy with oral glucocorticoids significantly reduced urinary level of 8-OhdG (a marker of oxidative DNA damage). It was noted that in patients who had recurrence of GO urinary level of 8-OhdG was high [47]. In the study by Akarsu et al. serum levels of serum level malondialdehyde (MDA, a product of ROS degradation of degrade polyunsaturated lipids) were higher in patients with GO, compared to controls and Graves' disease patients without GO. On the other hand, level of glutathione (GSH, a nonenzymatic antioxidant) was decreased in GO patients. Treatment with intravenous or oral methylprednisolone reduced MDA level. However, intravenous methylprednisolone induced more rapid therapeutic response and more rapid reduction in MDA level (in 4 weeks). Twelve weeks after the end of the treatment, clinical activity score and serum level of MDA were the same in both methylprednisolone-treated groups [48].

Treatment of Graves' disease with antioxidants is based on a premise of role of the OS in its' pathogenesis. A small trial using allopurinol and nicotinamide showed effectiveness of antioxidant treatment of mild and moderately severe Graves' ophthalmopathy [49].

Selenium is a trace element and is essential for selenoproteins synthesis where selenium functions as a redox centre. Some of selenoproteins like thioredoxin reductase and glutathione peroxidases play the key role in antioxidative defences. Most of the European countries are selenium deficient [50]. Previous clinical trials showed some effect of selenium on thyroid autoimmunity [51]. A recent large clinical trial evaluated effect of selenium on mild Graves' ophthalmopathy. Patients from several European countries were treated with sodium selenite in a dose of 100 *μ*g twice daily. Selenium treatment was associated with an improved quality of life and less eye involvement and slowed the progression of Graves' orbitopathy, compared to placebo [52].

## 6. Concluding Remarks

Evidence from previous studies suggests that oxidative stress plays a role in the pathogenesis of Graves' disease. *In vitro* and *in vivo* studies showed that antithyroid drugs and antioxidants influence parameters of oxidative stress both in retroorbital tissue and in the whole organism. However, until recently, all studies were small, nonrandomized, or uncontrolled, and large, controlled study was asked for [53]. Now, a large, randomized, controlled study proved that selenium supplementation significantly improves quality of life and reduces ocular involvement in patients with mild Graves' orbitopathy. Although it seems that antioxidative therapy will not play a major role in the treatment of Graves' disease, further trials are necessary to define its place as adjunctive therapy, or as the therapy for mild and moderate Graves' ophthalmopathy.
